# Pharmacy practitioners’ lived experiences of culture in multicultural Australia: From perceptions to skilled practice

**DOI:** 10.1371/journal.pone.0217673

**Published:** 2019-06-19

**Authors:** Jasmina Fejzic, Michelle Barker

**Affiliations:** 1 School of Pharmacy, The University of Queensland, Woolloongabba, QLD, Australia; 2 Griffith Business School, Department of Business Strategy and Innovation, Griffith University, Gold Coast, QLD, Australia; Georgia Southern University, UNITED STATES

## Abstract

**Objectives:**

The Code of Ethics of pharmacy practitioners in Australia recognises the obligation to provide care to patients in a culturally safe and responsive manner. The aim of this exploratory study was to examine how Australian community pharmacists understand and experience the concept of ‘culture’ in their everyday practice environment.

**Methods:**

Voluntary, semi-structured interviews were conducted at community pharmacy placement sites in South-East Queensland, Australia. Pharmacists were asked to recall an incident that evoked their cultural awareness during the course of their practice. The question stated, verbatim: “We are preparing our students to be pharmacists in a highly multicultural community. Can you think of an incident where you learnt something about another person’s culture or it made you more aware of your own culture? Please briefly describe the incident.” Reportable responses were collected from 59 of the 92 visited pharmacists. These responses were audio-recorded and transcribed. The data were collated and analysed through iterative, reflexive, thematic analysis using constant comparison.

**Results and significance:**

The responses provided a rich selection of lived experiences within Australian multicultural pharmacy practice, describing professional dilemmas, fears and the strategies employed to overcome practice challenges. Six main response categories were identified: (i) Language/communication challenges, (ii) Cultural attitudes and behaviours, (iii) Exposure to culture due to pharmacy location, (iv) Religion, gender, and age, (v) Prejudiced/perceived racist attitudes and discrimination towards ‘other’ cultures, (vi) Perceived ‘sameness’ of different cultures. The study has provided valuable insights into community pharmacists’ experiences of culture in their day-to-day professional practice, also highlighting the associated strategies used to maintain a high standard of practice. There is merit in ensuring that the pharmacy curriculum and professional development programs are designed to respond to the ethical obligation of pharmacists to practise in a culturally safe, responsive manner that acknowledges and incorporates the importance of culture, cultural differences and intercultural relations, while addressing culturally unique needs in a skilled and professional manner.

## Introduction

Australia is culturally and linguistically diverse (CALD). It has been enriched by the contribution of people from many nations and its policies remain committed to maintaining a culturally diverse, tolerant and open society where the language needs of CALD populations are addressed [1]. The oldest continuing human culture on earth, Aboriginal and Torres Strait Islander peoples, have lived on the continent for more than 50,000 years and their history and culture have also uniquely shaped the Australian nation [1,2]. The majority (75%) of Aboriginal and Torres Strait Islander peoples live in cities and non-remote areas, representing 3% of the total Australian population of 24.5 million [3,4]. Since colonisation, Australia has been an immigrant nation, with 28.5% of its current residents born overseas, and its population identifying with around 300 ancestries [1,5].

The Code of Ethics of pharmacy practitioners in culturally diverse Australia [2] recognises the obligation to provide care to the healthcare consumer (here referred to interchangeably as patient, client or customer [6]) in a culturally safe and responsive manner that acknowledges and incorporates the importance of culture and cultural differences, the assessment of cross-cultural relations, the expansion of cultural knowledge, and the adaptation of services to meet culturally unique needs [7]. Culturally safe, appropriate and competent care is a key strategy for improving access to services and health outcomes for all Australians [7]. Pharmacists also need to be aware of their own culture and beliefs, and be respectful of the beliefs and cultures of others, acknowledging how they may influence care needs, and avoiding any discrimination on the basis of these differences [2]. Good practice, underpinned by effective communication, involves cultural empathy, or an awareness of the cultural needs and contexts of all healthcare consumers to ensure good health outcomes are achieved, the values that are also inherent with the Pharmacy education [2,7]. More recently, the term ‘cultural intelligence’ or ‘cultural quotient’ has been used as a measure of how well a person can adapt and manage effectively in culturally diverse contexts [8]. A related term, ‘cultural competence’ is defined as “the set of behaviours, attitudes, and policies that come together to enable a system, agency, or professionals to work effectively in cross-cultural situations” [9]. Written in relation to health services for Indigenous people, but relevant more generally, it is argued that “developing and embedding cultural competence in health services requires a sustained focus on knowledge, awareness, behaviour, skills and attitudes at all levels of service, including at the operational or administrative service level, health practitioner level, practitioner-patient level and student-training level” [9].

Despite the many existing definitions of ‘culture’, the term itself remains rather confusing within the popular discourse. Hence, it is not surprising that ‘culture’ had the biggest spike as a search term in the Merriam-Webster dictionary, becoming their Word of the Year in 2014 [10]. In a traditional anthropological or sociological sense, the term ‘culture’ describes the collective behaviour patterns of a group of people, their way of life [11], including thoughts, communications, languages, practices, beliefs, values, customs, courtesies, rituals, interactions, roles, relationships, and expected behaviours of a racial, ethnic, religious or social group [12]. Most variations between cultures are underpinned by value systems, which lead people from different cultures to behave differently under similar circumstances [13,14]. Values constitute a society’s perceptions about what is polite versus impolite, or right versus wrong, shaping how we interpret behaviour and events, and providing the lens through which we find meaning [15]. Yet, in everyday life, we are often unaware of our own culture. It is simply not salient to us. The most obvious, ubiquitous, important realities are often the ones that are the hardest to see and talk about, yet in the day-to-day trenches of adult existence they can have life-or-death importance [16].

Culture was elegantly and famously described by Oberg through a familiar metaphor–that we only notice our own culture when we enter another, strange one, and begin to feel “like a fish out of water” [17]. With the cognizance that our own culture is as familiar, and undefinable as the air we breathe, how effective can one be as a health professional in recognising and working within one’s own cultural and societal context [13], while simultaneously engaging with other people’s complex cultural milieu? This actuality may be a good starting point in an investigation of how pharmacists manage culture in practice [2,7,15,18–23]. Research into intercultural interactions in pharmacy practice has been limited, mainly focusing on specific concerns or medical conditions [24–27], or on specific practitioner or consumer groups (e.g. refugees) [28–35], and rarely examining pharmacists’ overall understanding of culture in relation to healthcare provision [36–38]. Clearly, every aspect of pharmacy practice is affected by a pharmacist’s cultural competence, from basic communication to developing personal self-reflexivity, building rapport with colleagues and consumers, through to providing care [2,7,12,36]. The aim of present study was to examine how Australian community pharmacists understand and experience the concept of ‘culture’ in their everyday practice environment. One of the significant innovations of this study is that it has been framed, quite deliberately, at the broadly ‘grassroots’ level of the Australian community pharmacy, in order to capture a variety of potentially noteworthy perceptions, meanings, and practices within the multicultural pharmacy practice environment.

## Material and methods

Ethical clearance (PHM/03/10/HREC) was provided by the University’s Human Ethics Research Committee. Visits and interviews were conducted at 92 community pharmacy placement sites by the first-named author (while placements were in progress) in South-East Queensland, Australia as part of the regular Master of Pharmacy (MPharm) placement course activities. These placements (also known as practicums) involved ten days of community pharmacy placement over ten weeks (one day per week). Placement site visits were part of the Pharmacy School’s regular quality assurance to support preceptors and engage with them as educational partners through individual conversations. The preceptors (registered community pharmacists) were notified of these scheduled, one-off visits two weeks prior to the visit. Preceptors were advised of the aims of the visit. Primarily, the purpose of the meeting was to seek preceptors’ evaluation of the student’s performance during the placement. The research question relating to this study was the first question asked during the interview with preceptors (community pharmacists).

The preceptors were thanked for their contribution to this research. In the semi-structured interviews, each preceptor was asked to recall an incident that evoked their cultural awareness during the course of their practice. The question stated, verbatim: “We are preparing our students to be pharmacists in a highly multicultural community. Can you think of an incident where you learnt something about another person’s culture or it made you more aware of your own culture? Please briefly describe the incident.” This question was developed based on the lack of published evidence pertaining to the issue of how pharmacists address cultural issues in their practice. The importance of this research subject has also been emphasised in the Australian professional pharmacy guidelines [2,7,22]. Participation in the research was voluntary and the preceptors were free to withdraw. No identifying information was collected and preceptors’ responses were recorded in a de-identified format.

The qualitative research method comprised semi-structured interviews with community pharmacists to explore the single research question stated above. Semi-structured interviews were considered appropriate because they enabled exploration of the topic of culture, allowing an appropriate level of flexibility. This methodology enabled exploration of complex situations related to culture, and the interpretivist paradigm provided insight into the meaning of pharmacists’ experiences [39]. The responses were audio recorded and transcribed by the first author. The data were collated and analysed through iterative, reflexive, thematic analysis to characterise participants’ responses. The first author read the pharmacists’ responses on several occasions, made notes on important points, overall impressions, and emergent themes. The results were grouped into themes and sub-themes through an iterative approach, using constant comparison and reflective procedures. The second author reviewed the analysis process, themes and sub-themes, and suggested further integration to facilitate effective analysis and reporting, and to ensure rigour and trustworthiness of the research.

## Results and discussion

Reportable responses to the culture question were collected from 59 of the 92 visited preceptors, since 22 were ‘too busy’ to participate in the research component of the interview, while 11 of the remaining 70 preceptors chose not to comment on the ‘culture question'. The researchers noted that this experience was congruent with the results of the recent systematic review reporting that the most common barriers to participation in pharmacy practice research are a lack of time and workload [40]. In some instances, participants had to conclude the interview and recommence their work before the interviewer sought basic demographic details, which is why demographic data was collected for only 45 participants. This sub-cohort of practitioners practiced pharmacy for 13.5 years on average, and 59% were female, which, coincidentally, matched the gender distribution of Australian pharmacists [41]. The rationale for asking the research question arose as a means of obtaining a succinct ‘snapshot’ of the pharmacy practice events that pharmacists associate with culture. Pharmacists’ responses varied in scope, quite candidly, intricately, and reflectively detailing a rich selection of their lived experiences within Australian multicultural pharmacy practice. Some also described ongoing professional dilemmas, fears, and the strategies they employ to overcome any concerns. The respondents' observations were classified into six categories (number of mentions in parentheses), namely: (i) Language/communication challenges (36), (ii) Cultural attitudes and behaviours (22), (iii) Exposure to culture due to pharmacy location (13), (iv) Religion, gender, and age (12), (v) Prejudiced/perceived racist attitudes and discrimination towards ‘other’ cultures (6), (vi) Perceived ‘sameness’ of different cultures (4). Some individual respondents mentioned several issues classified across the corresponding themes. Overall, the most common cultural associations were language and communication challenges, and cultural attitudes, beliefs and behaviours. Each theme is discussed in detail, illustrated by exemplar quotes from participants.

### Theme 1: Language/communication challenges

The most commonly reported theme addressed communication issues with customers from CALD backgrounds, and strategies Pharmacists used to ameliorate language barriers. This important aspect of contemporary, patient-focused professional practice, has also been reported in the literature as one of the main challenges faced by health professionals regardless of the discipline [12,15,21,24,33,42–44]. Pharmacists described language and communication barriers as prevalent when their customers have a non-English speaking/CALD background. The comment by Pharmacist 10: “Many communication and language barriers exist” was echoed by the majority of participants. Some reflected on the additional challenges of accented English: “Trying to understand people with an accent can be very hard” (Pharmacist 42) and commented on the practice difficulties: “It can be difficult to communicate with some customers, but I try and give them the information they need.” (Pharmacist 38). Pharmacists invested considerable effort into overcoming the language barriers and ventured well beyond the expected scope of practice to attempt to communicate effectively with patients. For example, Pharmacist 55 who had a Spanish-speaking clientele, reported: “I have a lot of foreign customers, so I had to brush up on my Spanish”. Pharmacist 54 even developed signage in customers’ language: “I have compiled signs in Chinese for my customers because we have a big Asian community”. Pharmacists also regularly trialled any available means, including non-verbal behaviour, to overcome communication barriers, with Pharmacist 8 observing: “Sometimes you communicate via gestures when the language proficiency is missing”.

Australia’s Translating and Interpreting Service (TIS National) provides access to phone and on-site interpreting services in over 150 languages for people who do not speak English and for agencies and businesses that need to communicate with their non-English speaking clients [45]. The service is available to Australian pharmacies to facilitate communication with non-English speaking holders of a Medicare card about the proper use of medications dispensed through the Pharmaceutical Benefits Scheme (PBS) [46]. Still, the optimal, professional and ethical supply of non-PBS items to people with poor English proficiency remains unaddressed [2,7]. This service does not provide assistance to non-residents and tourists, populations that Australian pharmacists commonly encounter in practice [5,12,47]. Interestingly, only two participants mentioned the free translating and interpreting service^46^ in their responses. Achieving effective communication between the healthcare provider and the consumer is the central requirement of successful pharmacy practice globally [2,7,12,23,29,42,48], and further research is needed to ascertain the extent to which communication challenges are addressed in Pharmacy education curricula as well as pharmacy practice. The current study also pointed to pharmacists’ concerns and doubts about whether they were understood fully by their clients. For example, Pharmacist 58 reflected: “Language problems are difficult, and I am not sure if patient really understands me, either regarding their drugs or side effect.”. Clearly, any miscommunication regarding the appropriate use of medicines, as well as warnings about their side effects, can cause harm to the patient and result in suboptimal treatment. Some participants used a family member of the patient as an interpreter, mentioning potential ethical issues around privacy and understanding of the pharmacist’s directions. Pharmacist 13 was not alone in admitting frankly: “If a customer does not speak English I sometimes have to speak through their partner. I am questioning what the privacy issues are here, and I am unsure if the partner is actually understanding, and what is the customer understanding”. These findings indicate the scope for additional education and ongoing professional development for pharmacists around the use of regulated, high quality telephone interpreting services in order to comply with the national code of conduct, professional practice guidelines, and regulations [2,7].

Some pharmacists were solution-focused in their answers, describing how they were “…using their multicultural staff’s skills in order to address any language barriers” (Pharmacist 1), and how “We had South African customers and our student speaks Afrikaans, so they were able to communicate” (Pharmacist 29). It may be useful to have someone in the pharmacy speaking the customer’s language (as opposed to no one), however, as professionals, pharmacists should be using professional interpreters [1,2,7,46] rather than using staff or students untrained in professional interpreting techniques [1,2,7,46]. Naturally, it would be appropriate for pharmacists with a sound command of a second language to communicate directly with the customer, however, one pharmacist observed that even “…when the pharmacist or assistant speak the language that the customer speaks…language barriers still occur” (Pharmacist 21).

Participants with CALD backgrounds noted that they were already aware of the impact of culture in their practice, observing: “I am from an Asian country, so I am very aware already of cultural differences, and open to cultural diversity” (Pharmacist 17), and stating: “I am from a Middle-Eastern culture, so I am largely aware of the cultural issues and super sensitive to other cultures” (Pharmacist 59). While these may be salient self-perceptions, it cannot reasonably be concluded that CALD status automatically guarantees cultural competence [2,7,36], since there is more complexity surrounding the variable of culture than simply being cognisant of one’s own ‘otherness’ within the mainstream cultural context. ‘Self-reference criterion’ refers to the unconscious reference point by which an individual understands and relates to other people only in terms of their own cultural values, similar to ‘projective cognitive similarity’ which is an assumption that others think, reason, and judge in the same way as ourselves [13].

### Theme 2: Cultural attitudes and behaviours

The second most reported association with culture related to encountering various cultural traits, beliefs, attitudes, and foreign pharmacy practices. Pharmacists tried to remain “open to cultural differences, be patient, because the patient needs to understand medicines and side effects, and we would not want people to go away uneducated” (Pharmacist 44). They dwelt on deeper meanings and associations that limit communication, observing that: “Cultural barriers may also mean that patients don’t want to hear what they’ve been told, even when they understand the language.” (Pharmacist 10). Stereotypes, as a type of schema, can be defined as “the assumptions that every member of a society or subculture has the same characteristics or traits, without regard to individual differences” [13] and are quite common in society and workplaces [20,25], with community pharmacy being no exception. Participants mentioned a variety of cultural characteristics they encountered regularly in the workplace, and displayed a high level of cultural understanding and sensitivity [2,7,29].

“It is very challenging when some cultures (e.g. those from the Former Yugoslavia) take things so very personally.” (Pharmacist 5).

“Koreans don’t pick up on emotional cues of others, so it can be difficult for pharmacists to express empathy, because they’re ‘cool’.” (Pharmacist 7).

“We have diverse customers, for example Germans can be quite blunt sometimes, and this can be misconstrued for being rude.” (Pharmacist 57).

“It happens all the time…Japanese tend to say ‘yes’ to every question, even if they don’t understand what our Japanese pharmacist is saying.” (Pharmacist 59).

Despite the customer and the pharmacist having the best intentions, culture sometimes complicated interpersonal interactions in practice, a point noted in the literature [36]. Sensitive and uncomfortable cultural ‘clashes’ can be unavoidable, due to the specific individual experiences and histories associated with the cultural heritage of both staff and customers [29]. The following quotes illustrate incidents where stereotyping is apparent [49].

“One’s name can elicit cultural presumptions, for example, people tell me ‘oh my god, you’re Middle Eastern, I love Middle Eastern food’… Also, people prefer to be spoken to informally rather than in an overly polite manner which can be seen as too distant–e.g. when I say ‘sir’ it can be offensive to some people, which is amplified further because they think I am an Arab.” (Pharmacist 16).

Pharmacists highlighted how pharmacy practices varied between countries and how cultural differences in the use of medicines had consequences that could cause frustration to the pharmacist and/or the patient, affecting patient care:

“In New Zealand, they keep repeats in the same pharmacy that they go to so when they move to Australia they expect the same thing, which causes confusion and their repeats to be misplaced because they’re used to the pharmacy keeping them for when they return.” (Pharmacist 44).

“We had an Asian customer with osteoporosis and the doctor advised her to choose her own over-the-counter medication, so pharmacist had to assist the customer and help comfort her as she was freaked out for having to do it herself.” (Pharmacist 53).

“A lot of medicines around the world don’t need prescription to the degree that we do in Australia, which can cause frustration.” (Pharmacist 56).

The Australian government-subsidised PBS system was referred to sarcastically as ‘great’ by one pharmacist, specifically in relation to Germans, who “live here and must pay the full price for the medicines.” (Pharmacist 47). It is unclear from the transcripts why the pharmacist singled out Germans in this instance since other foreign nationals are pay the full price for prescription medicines, unless they have Permanent Resident status.

Several pharmacists appreciated the way in which Pharmacy students from CALD backgrounds were open to sharing information about their own cultural background in a way that enriched practice. Interestingly, when thinking about how cultural awareness and how cultural differences may impact on pharmacy practice, one pharmacist referred to a student who came from another culture. He commended the student for her self-awareness and cultural awareness, both of which are qualities that are highly desirable in Pharmacy students [20,50,51]. Pharmacist 34 explained: “Our student is Egyptian Muslim and she has made us more aware of her Muslim culture and historic background. Her nature is very open–she comes from the perspective of the ‘other’ and uses it to her advantage”. In the same vein, Pharmacist 36 stated: “…this student is open about her different culture, she can bring a different point of view about healthcare to our practice”.

In one instance, Pharmacist 56 perceived some pharmacy students’ ‘practice culture’ as unfavourable and counter-cultural (compared to mainstream culture), because the students lacked what the pharmacist considered ‘passion’ for Pharmacy, focusing instead on the business aspects of being a pharmacist: “We get students from Arab countries who are only really interested in the business of pharmacy, there is no passion, but a point of view that is very different from that of the Australian healthcare”.

### Theme 3: Exposure to culture due to pharmacy location

Some associated the community pharmacy’s location with customers’ diverse cultural backgrounds, for example, Pharmacist 38 commented: “We are close to a university, so we are dealing with many different cultures”. Similarly, Pharmacist 49 observed: “Our pharmacy is close to the airport–we see diversity every day”. In addition, some pharmacists highlighted subcultural differences within Australia, particularly within rural and semi-rural areas: “Even Australians in rural areas are different amongst themselves. Every day is a learning experience–to overcome prejudices and my own reactions to people different from ourselves–I bring all my skills.” (Pharmacist 32), illustrating the commitment to life-long learning and critical reflection on personal prejudices, as well as commitment to intercultural competence development [2]. Interestingly, as Oberg reminded us, it is in the contrast that culture becomes more salient. Several pharmacists perceived the lack of cultural diversity itself in their clientele as a reminder about culture, noting they were: “Working in a highly Australian middle-class suburb that is very white–which is a cultural difference too.” (Pharmacist 33), and that “Northside Brisbane is a bit less culturally diverse.” (Pharmacist 7). The pharmacists’ surprise at the lack of cultural diversity is understandable given the number of immigrants, tourists and international students who chose to live, visit or study in Australia, a multicultural nation well connected to the rest of the world by air, with education being its third-largest export and the largest services export [5,52].

### Theme 4: Religion, gender, and age

Diversity, in terms of religion, gender, and age are commonly encountered subcultural elements in any Australian workplace [1,5,7,37,53–55]. Therefore, it was no surprise to see various aspects of pharmacy practice being closely associated with customers’ religion and the interconnectedness of religion and culture. Exemplar statements from participants referred to religious diversity, particularly in relation to Muslim customers, including the following responses:

“Muslim patients–when they are taking tablets or capsules and I need to know if they are gelatine free (halal).” (Pharmacist 6).

“Muslim couple came in, the prescription was for the wife, and the expectation was to direct questions to the husband and do the couple counselling with him involved.” (Pharmacist 8).

In one case, Pharmacist 11 commented on the ease with which he/she conversed with a family of Muslim faith, stating: “I have been surprised with how easy it’s been to interact with a Muslim family…it is always important to ask questions, then everything can feel comfortable at the end”. Another pharmacist expressed surprise about learning more about their own culture from customers: “Gelatine-based capsules contain a certain type of gelatine. I am Muslim, as are some other staff too. This Muslim customer wanted ‘halal gelatine capsules.”. Even though we share the same culture, I was surprised with the question as I had never come across it before.” (Pharmacist 12). In addition, Pharmacist 21, compared his experience in meeting the needs of Jewish customers, commenting: “Kosher medicines in the UK is a big thing. I am unsure if this is the case in Australia”.

'The importance of suitable Pharmacy education that enables competent intercultural communication and the need to avoid making assumptions was abundantly evident [12,28,36,37]. These findings were highlighted in the context of pharmacies needing to operate as successful businesses: “A person is dressed in a ‘full burka’. You must make sure that you smile and you are friendly and ensure that you are welcoming of their culture as this may mean that there is a return visit to your pharmacy.” (Pharmacist 27). In addition, the lack of availability of pharmacy services during religious holidays meant that: “Not everyone celebrates Easter, so when we close over the Easter period not all customers understand or are happy with this, but they have to understand that their culture may not believe in Easter but others do.” (Pharmacist 40).

The issue of gender was also noted. For example, Pharmacist 13 stated: “Some cultures do need more sensitivity and discretion around female issues”. While two other Pharmacists noted clients’ gender preferences: “Sometimes a male customer asks for a male pharmacist.” (Pharmacist 11 and 22). another highlighted a customer’s assumption that the Pharmacist would be male: “A male customer in his sixties asks ‘where is the pharmacist?’ because in his experience (the) pharmacist is normally a middle-aged man.” (Pharmacist 30). A positive experience was described in relation to: “…older customers from certain cultures (e.g. Greek, Italian) who sometimes have good English also bring in ethnic food for the pharmacy staff.” (Pharmacist 29). Culture was clearly perceived as a rapport-building factor between pharmacy staff and customers from well-established immigrant cohorts in Australia such as those from Italy or Greece.

### Theme 5: Prejudiced/perceived racist attitudes and discrimination towards ‘other’ cultures

While the area of healthcare is not immune from the inevitable echoes of prejudice and racism [12,56], it is still somewhat confronting to uncover examples of such phenomena within one’s own profession in relation to pharmacists, students and customers. One pharmacist stated that “I am Asian and fully aware of the multicultural diversity and the existing racism in the community”. (Pharmacist 4), while another observed that “Culture is a massive issue. I am Middle-Eastern and I am sometimes told that positions are filled when they’re not” (Pharmacist 16). Another pharmacist shared: “There are racist customers. I am Asian, as you can see, so I know this … it is rare, but sometimes customers will ask for an ‘Australian’ to serve them” (Pharmacist 17). Another response referred to customers’ comments that were patronising, if not racist, towards pharmacy staff: “People will say that ‘little Asian one’ for a pharmacy assistant” (Pharmacist 20). Likewise, one pharmacist referred to racist attitudes towards pharmacists of Asian cultural origin by customers who are war veterans: “We have Asian pharmacists and war veteran customers–which can be a bit of a problem. We have an RSL (Returned Services League) nearby, so these ‘things’ happen.” (Pharmacist 18).

One pharmacist noted that: “International students can be more difficult to supervise, yet they may go back to their own country after uni, still taking an Australian citizenship spot” (Pharmacist 35). This line of reasoning is difficult to follow, but may be explained in part through Social Identity Theory (SIT) according to which a person’s self-concept can be centred on the extent to which they identify with particular social groups, and the associated value and emotional significance [57]. An ‘ingroup member’ is perceived to be like ourselves (e.g. same ethnicity, social class, profession). Conversely, an ‘outgroup member’ is different from us in a way that we do not value (e.g. different ethnicity, social class, profession) [57]. Apart from Pharmacist 35’s supervisory frustrations, it may be that the pharmacist feels that the training provided is ‘wasted’ on a ‘foreign’ student (‘outgroup’ member) who intends to return to their home country after graduation, as well as a misinformed view that international students compete with Australian students (‘ingroup’ members) for Pharmacy university places when international and local/domestic students in fact have separate, independent admission pathways in Australia [58]. While expressed by a minority, these responses provide insight into prejudiced attitudes in pharmacy practice and highlight the need to prepare students for possible prejudiced reactions they may encounter. This theme also foregrounded the need for Pharmacy education and ongoing professional development for pharmacists in the related areas of intercultural awareness and intercultural competence [2,9], in line with Australia’s National Anti-Racism Strategy [59].

### Theme 6: Perceived ‘sameness’ of different cultures

Several pharmacists noted that “All customers are the same.” (Pharmacist 35), so they “Try to treat all situations the same”. (Pharmacist 37) and, in the process “Use the same skills.” (Pharmacist 22). It could be possible that these pharmacists were advocating for a patient-centred approach. Given the specific mention in the Code of Ethics about addressing linguistic and cultural diversity, these pharmacists also seemed to be overlooking individual patient differences, which is contrary to demonstrating cultural sensitivity underpinning professional intercultural competence, as per the Code [1,2,7,19,50]

Despite only one respondent stating that they “Don’t really have to deal with customers from other cultures.” (Pharmacist 50), this response is included as an outlier. According to Osborne and Overbay, outliers are: “a data point that is far outside the norm for a variable or population” [60]. If outliers are due to error or intentional misreporting they might be removed, whereas, in this case, they might be taken into consideration if they are perceived to be of interest for additional inquiry. This response is important since it elegantly reminds the reader that the phenomenon of culture can be entirely misperceived by a health professional. As it is improbable that a practicing Australian pharmacist has not dealt with at least some aspect of ‘culture’ in the course of a single day, one is left to ponder what might underpin this response. The respondent may have been disinterested in the topic, or genuinely did not perceive cultural differences in practice.

Future research could aim to include a broader, generalisable, cross-section of the profession, including pharmacists from a range of years of professional experience, demographics, geographical locations, as well as practice environments. Overall, the conclusions reached must be considered in light of particular limitations, especially in relation to sample size. As an exploratory study, the qualitative interviews provided some opportunity for pharmacists to reflect about an area of investigation that has not received previous attention. The research points to possible future directions for research and ongoing professional development of pharmacists. For example, specific continuing professional development modules on working effectively with CALD customers and capability building in the area of intercultural competence, could be trailed and evaluated. Research is also needed to establish the extent to which interpreters are utilised in pharmacy practice. Effective pharmacy services delivery would also need to be underpinned by consistency in definitions, understanding and implementation of cultural competence in pharmacy practice [61]. The impact of locale on practitioners’ experience of cultural and linguistic diversity amongst colleagues and clients is also well worth further exploration. While there was no mention of working with Aboriginal and Torres Strait Islander customers in particular, it is important that future research explores pharmacists’ understanding of the needs of this subgroup within multicultural Australia. If we are to enhance pharmacists’ cultural competence [62]^,^ we need to capture and document their experiences in communicating with and providing pharmacy services to people from culturally and linguistically diverse backgrounds.

## Conclusion

The study has provided invaluable insights into community pharmacists’ experiences of culture in their day-to-day professional practice in multicultural Australia. It also highlighted the associated challenges and strategies they use to maintain a high standard of practice in intercultural situations in the workplace. Clearly, many pharmacists are attuned to the complexities involved in meeting the needs of clients, peers and pharmacy students from CALD backgrounds. For others, culture was not particularly salient. The exploratory study indicates there is merit, therefore, in ensuring that the Pharmacy curricula and professional development programs are designed to respond to the ethical obligation of pharmacists to practise in a culturally safe and responsive manner that acknowledges and incorporates the importance of culture, cross-cultural relations and differences, and addresses culturally unique needs in a skilled and professional manner [7]. To ignore the complex influence of culture on practice, would, to borrow Oberg’s metaphor [17], constitute ignoring the very medium in which we swim.
